# ASO Author Reflections: Racial and Sex Differences in Genomic Profiling of Intrahepatic Cholangiocarcinoma

**DOI:** 10.1245/s10434-024-16263-z

**Published:** 2024-09-23

**Authors:** Diamantis I. Tsilimigras, Timothy M. Pawlik

**Affiliations:** Department of Surgery, Division of Surgical Oncology, The Ohio State University Wexner Medical Center and James Comprehensive Cancer Center, Columbus, OH USA

## Past

Intrahepatic cholangiocarcinoma (iCCA) is an aggressive primary liver tumor clinically and biologically distinct from other liver or biliary tract cancers.^1^ Up to 50% of iCCA patients may harbor potentially actionable genomic alterations, with *IDH1* mutations and *FGFR2* fusions currently considered the most promising targets.^1^ Both *FGFR* and *IDH1* inhibitors are currently approved by FDA for populations with the respective alterations.^2^ Historically, however, the enrollment of Black and other non-White patients in precision oncologic studies has been low, thereby limiting our understanding of the biologic/genomic heterogeneity of the disease in diverse populations.^1^ In addition, prior studies have reported racial and sex disparities in the incidence of iCCA and the outcomes for patients with iCCA,^3^ yet variations in genomic profiling of iCCA that may be contributing to the disparate outcomes among different patient populations have not been thoroughly examined. To this end, the current study sought to comprehensively assess genomic alterations of patients with iCCA based on race and sex using a large genomics dataset.^4^

## Present

Of the 1068 patients with iCCA who had available sequencing data in the American Association for Cancer Research GENIE project database,^5^ 71.9% were White, 8.4% were Asian, and 5.1% were Black (other/unknown, 14.6%). The male-to-female ratio was 1:1. The most commonly mutated genes among all the iCCA patients were *TP53* (21%), *IDH1* (20%), *BAP1* (17%), *FGFR2* (16%) and *CDKN2A* (14%). The *IDH1/2* and *FGFR2* mutations were mutually exclusive, whereas the *FGFR2* gene was frequently co-mutated with *BAP1.* In addition, the *IDH1* gene was frequently co-mutated with *PBRM1* (all *p* < 0.05). The *IDH1* and *PBRM1* mutations were more frequent among White patients than among Asian or Black patients with iCCA (*IDH1*: 20.8% vs. 17.8% vs. 5.6% [*p* = 0.021]; *PBRM1*: 14.5% vs. 11.4% vs. 0% [*p* = 0.015]). In contrast, the *FGFR2* gene tended to be more frequently altered among Black versus Asian or White patients with iCCA (27.8% vs. 15.6% vs. 16.1%; *p* = 0.08). In addition, female patients more frequently had mutations in *IDH1* (23.3% vs. 16.0%), *FGFR2* (21.0% vs. 11.3%), and *BAP1* (23.4% vs. 14.5%) genes than male patients, whereas *TP53* mutations (24.3% vs. 18.2%) were more prevalent among male than among female individuals with iCCA (all *p* < 0.05). Significant variations in other genomic alterations also were noted across races and sexes.

## Future

Next-generation sequencing showed marked variations in the genomic profiling of iCCA patients based on race and sex. Although the percentage of non-White patients with sequenced iCCAs was low, the data suggested that a large proportion of iCCA patients might be candidates for targeted therapies, highlighting the need for mutational analysis for all patients irrespective of race or sex. The findings of the study also demonstrated that *FGFR2* and *IDH1* alterations frequently co-occur with other mutations among iCCA patients. Future studies should focus on investigating co-mutation patterns, their impact on long-term prognosis, and their association with response to certain targeted therapies. In addition, future clinical trials should include diverse patient populations with iCCA to identify distinct targetable genetic drivers of disease among different patient cohorts.
